# Structured Reporting in Radiological Settings: Pitfalls and Perspectives

**DOI:** 10.3390/jpm12081344

**Published:** 2022-08-21

**Authors:** Vincenza Granata, Federica De Muzio, Carmen Cutolo, Federica Dell’Aversana, Francesca Grassi, Roberta Grassi, Igino Simonetti, Federico Bruno, Pierpaolo Palumbo, Giuditta Chiti, Ginevra Danti, Roberta Fusco

**Affiliations:** 1 Division of Radiology, Istituto Nazionale Tumori IRCCS Fondazione Pascale—IRCCS di Napoli, 80131 Naples, Italy; 2 Department of Medicine and Health Sciences “V. Tiberio”, University of Molise, 86100 Campobasso, Italy; 3 Department of Medicine, Surgery and Dentistry, University of Salerno, 84084 Salerno, Italy; 4 Division of Radiology, Università degli Studi della Campania Luigi Vanvitelli, 80127 Naples, Italy; 5 Italian Society of Medical and Interventional Radiology (SIRM), SIRM Foundation, 20122 Milan, Italy; 6 Department of Applied Clinical Sciences and Biotechnology, University of L’Aquila, 67100 L’Aquila, Italy; 7 Department of Diagnostic Imaging, Area of Cardiovascular and Interventional Imaging, Abruzzo Health Unit 1, 67100 L’Aquila, Italy; 8 Division of Radiology, Azienda Ospedaliera Universitaria Careggi, 50134 Florence, Italy; 9 Medical Oncology Division, Igea SpA, 80013 Napoli, Italy

**Keywords:** radiology, standardization, quality, lexicon, tumor

## Abstract

Objective: The aim of this manuscript is to give an overview of structured reporting in radiological settings. Materials and Method: This article is a narrative review on structured reporting in radiological settings. Particularly, limitations and future perspectives are analyzed. RESULTS: The radiological report is a communication tool for the referring physician and the patients. It was conceived as a free text report (FTR) to allow radiologists to have their own individuality in the description of the radiological findings. However, this form could suffer from content, style, and presentation discrepancies, with a probability of transferring incorrect radiological data. Quality, datafication/quantification, and accessibility represent the three main goals in moving from FTRs to structured reports (SRs). In fact, the quality is related to standardization, which aims to improve communication and clarification. Moreover, a “structured” checklist, which allows all the fundamental items for a particular radiological study to be reported and permits the connection of the radiological data with clinical features, allowing a personalized medicine. With regard to accessibility, since radiological reports can be considered a source of research data, SR allows data mining to obtain new biomarkers and to help the development of new application domains, especially in the field of radiomics. Conclusions: Structured reporting could eliminate radiologist individuality, allowing a standardized approach.

## 1. Introduction

As stated by the American Recovery and Reinvestment act, and the Health Information Technology for Economic and Clinical Health act, the best medical practice should be based on structured data, in order to improve patient clinical outcomes [1,2]. In this view, radiology reports, as health record elements, should be conceived in a structured report (SR). In fact, habitually, radiology reports are free text reports (FTRs), based on a descriptive communication.

The free text report has [1] a descriptive section, which describes the relevant findings, according to the clinical information and the diagnostic question to be answered by the imaging study; the description of relevant incidental findings, i.e., not related to the clinical symptoms/radiological question; and, eventually, the description of “irrelevant” findings. It also has [2] a conclusive section, with diagnosis, differential diagnosis, and an eventual recommendation for additional (imaging) studies or diagnostic tests. Unexpected relevant findings, acute findings, or findings requiring urgent/immediate therapeutic action must be communicated immediately to the referring physician and this communicative act must be documented in the radiological report.

Many radiologists, despite the FTR, use a form of SR in their report, i.e., they use widely accepted classification systems (fractures, pancreatitis, TNM, etc.) [3,4,5,6,7]. In several centers, the radiologist’s FTR is adapted to the preferences of the referring physician, based on the experience of multidisciplinary team discussions, in which the radiologist is involved. Here, the radiologist “learns” what is crucial for reporting consistently and quantitatively, leading to uniform communication with the physician and, thus, contributing to more appropriate diagnostic and therapeutic management. Since, in accordance with what is reported in a radiological report, a multidisciplinary team establishes the therapeutic approach, it is evident that the communication of radiological data is crucial to avoid communication errors or poor patient management [3,4,5,6,7]. From this point of view, the SR should be considered as a tool to reduce radiological error [3,4,5,6,7]. However, the issue of whether all radiological studies should have a SR remains open [1,8,9,10,11,12,13,14,15].

**Figure 1:**
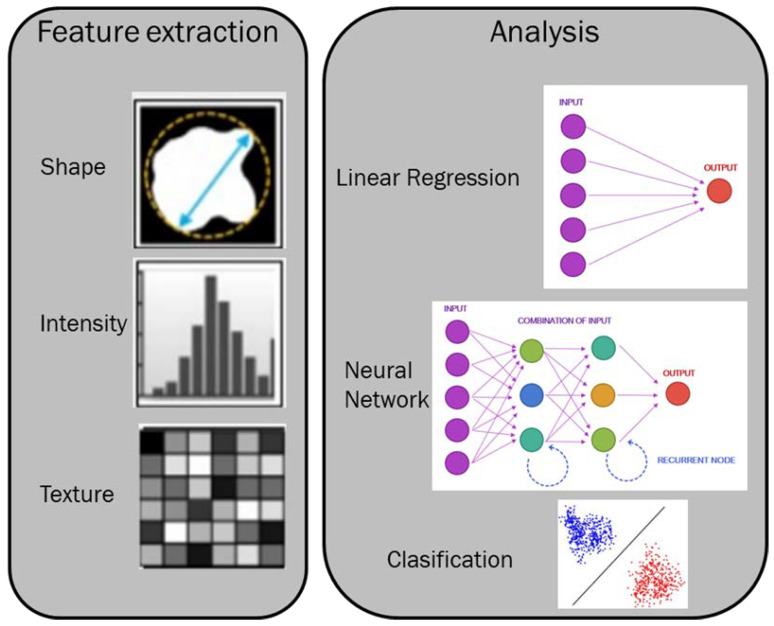
Representation of feature extraction and analysis in a radiomics process.

As stated by the European Society of Radiology’s (ESR) paper on SR [1], the three main aims in shifting from FTRs to SRs are quality, datafication/quantification, and accessibility. With regard to quality, it is due to standardization. The opportunity to employ a “structured” template in order to report all the relevant data for a radiological study, allows us to link the imaging findings with the other clinical features, guiding us towards personalized medicine. With regard to accessibility, since radiological reports are an abundant resource for data research, these allow automated data mining. This process could help to develop new biomarkers for potential new application domains [1,16,17,18,19,20], such as a radiomics field [21,22,23,24,25,26,27,28,29,30]. Radiomics is an innovative field of imaging research and, thanks to the numerical data obtained from radiological studies (Figure 1), it is possible to obtain a more objective evaluation of the patient’s status. In this context, SR could help radiomics analysis.

## 2. Method

This article is a narrative review on SR in radiological settings. Particularly, its limitations and future perspectives are analyzed.

## 3. Description

As stated by Weiss et al. [31], the SR could be “structured” in three levels, as follows:

I. At the first level, a SR is subdivided into sections and subheadings. Now, all radiological reports have these forms, including sections for clinical data, study protocols, radiological findings, and conclusions to emphasize the main radiological features.
II. At the second level, the report is organized, explaining all the relevant specific disease findings.
III. At the third level, the report includes a standard lexicon.

The SR’s advantages are due to several main reasons, as follows: quality and accuracy, accessibility, workflow simplification, automatization, retrievability, up-to-date electronic patient records (EPRs), economic benefits, education, and standardization between radiological centers [32,33,34,35,36,37,38,39,40].

Accuracy and quality are critical issues. The SR could support radiologists in using an appropriate lexicon and, so, could prevent unclear and prolix reports [1].

## 4. Perspectives and Clinical Settings

Since SRs are “structured” in sections, which include clinical history, indication, technique and study protocol, imaging findings, and conclusions, the possibility of connecting the template and a patient’s electronic file, allows an automatic export of all the available patient data. This process improves the radiologists’ workflow, ensuring that they know all the data about the patient. Therefore, the opportunity to assess radiological data with clinical data, including patients’ anthropometric data, family or previous history of cancers, influencing diseases and risk features, and histopathological study results, allows us to obtain robust datasets, which could be evaluated for both epidemiological and statistical studies, but also to build radiomics analyses [41,42,43,44,45,46,47,48,49,50,51,52,53]. Considering this point of view, the additional value of genomic data can be used to develop radiogenomic models, which are useful for acquiring the highest level of personalized risk stratification and for the advanced precision medicine process. These models are attracting a great deal of attention in the field of early diagnosis for lung and breast cancer, stratifying patients into different risk categories in order to build a diagnostic study protocol suitable for individual categories.

Furthermore, the possibility of sharing the examination technique and reporting the technical parameters used during the examination allows for a dual purpose. Firstly, it allows us to improve the examination protocol because of the standardization between the different centers. Secondly, it allows us to compare the results obtained between centers. In fact, the protocols of different studies could influence the results and, therefore, decrease the possibility of comparing these results, of the diagnostic accuracy, and of the reproducibility of the data. For example, in computed tomography (CT) studies, during oncological patient surveillance, differences in the studies’ parameters and the algorithms employed are central features that can cause variability in dimensional assessment [54,55,56]. Therefore, slice thickness and other protocol-related features, such as the reconstruction kernel and the field of view, should remain unchanged throughout patient follow-up. The CT variability decreases with standardized protocols. In fact, assuming that standardized protocols allow continuity and consistency, they improve precision and accuracy in CT image quantification in the key areas of optimization—assuming that standardized protocols should allow for an improvement in patient safety (e.g., radiation dose reduction), contrast optimization, and image quality.

In magnetic resonance imaging (MRI), data sharing regarding the type of study protocols used—such as conventional morphological (i.e., T1 or T2 weighted (W)) and functional sequences (diffusion weighted imaging (DWI) and dynamic contrast enhancement (DCE)), and the contrast agents employed (interstitial or hepatospecific for liver study)—should normally be undertaken. The sharing of study protocols is crucial, since one of the main challenges of MRI is the lack of standardization. Similar protocols need to be performed with a view to the reproducibility of the data [57,58,59,60,61,62,63,64].

**Figure 2:**
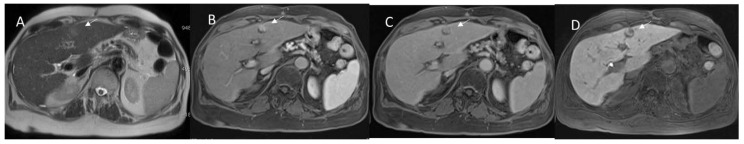
HCC EOB-MRI assessment. The lesion shows (arrow) hyperinthense signal on T2-W: (**A**) sequences, (**B**) arterial hyperenanchement during arterial phase of contrast study, (**C**) wash-out appearance during portal phase, and (**D**) hypointense signal during hepatospecific phase.

**Figure 3:**
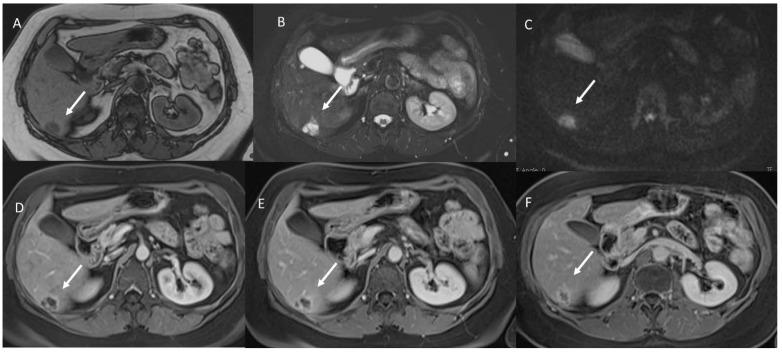
Colorectal mucinous liver metastases, assessed with non-liver-specific contrast agent. The lesion (arrow) shows hypointense signal in T1-W: (**A**) sequence; (**B**) very high hyperintense signal in T2-W; (**C**) restricted diffusion; and targetoid appearance during (**D**) arterial, (**E**) portal, and (**F**) late phase of contrast study.

With regard to contrast agents in MRI liver studies, today, two types of agents could be used. According to the different phase of patient management, the study protocols can include the possibility to administrate a liver-specific contrast (in pre-surgical settings) and a non-liver-specific contrast (in the characterization and staging phases). Liver-specific contrast agents can also be used to assess functional liver failure in both patients with hepatocellular carcinomas (HCC) (Figure 2) and in liver metastatic patients (Figure 3). Therefore, to understand the pattern of the lesion during the study of the contrast medium and the functionality of the liver parenchyma, the radiologist should clarify the type of agent used. Furthermore, the contrast agent is a drug and could cause a reaction, so these data should be reported in a SR.

**Figure 4:**
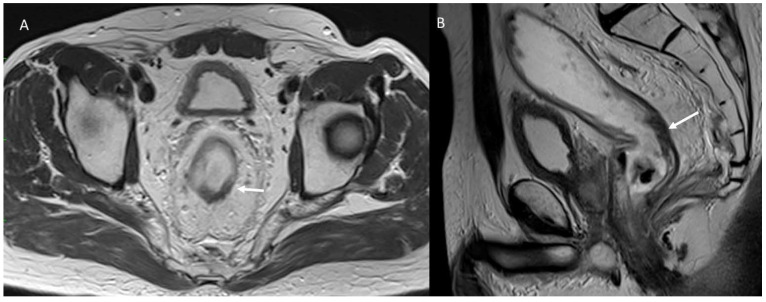
MRI assessment post n-CRT treatment: fibrotic response in T2-W axial (arrow) (**A**) and sagittal plane (arrow) (**B**).

At the second level, the report is based on the presence of a “findings” section, organized with subheadings. With this “structure”, it is possible to report all lesion-relevant data. For example, during MRI rectal cancer staging [65,66], the templates should report all the relevant issues on primary lesions and nodal status, such as the circumferential resection margin’s (CRM) involvement, extramural venous invasion (EMVI), and tumor deposits, in order to define the proper patient treatment. For MRI rectal cancer restaging, all relevant issues are re-assessed [65,66] to define the treatment response and the proper patient therapeutic approach (Figure 4), (i.e., total mesorectal excision, versus the “wait and watch” approach) [67,68,69,70,71,72,73,74].

**Figure 5:**
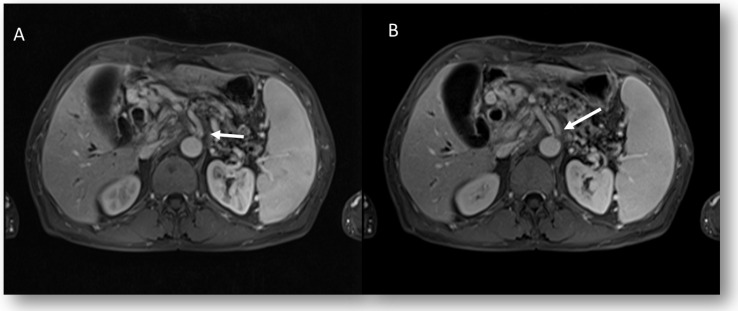
Pancreatic cancer patient. MRI staging assessment (arterial (**A**) and portal (**B**) phase of contrast study). The arrows show right hepatic artery origin from the superior mesenteric artery.

In the assessment of pancreatic cancer [75], the multidisciplinary team should make the choice concerning the lesion resectability following the acquisition of a complete staging [76,77], based on CT and MRI studies [75,76]. The lesion resectability is related to the following three different features: anatomical (A), biological (B), and conditional (C). Anatomic features include tumor contact with the superior mesenteric artery and/or celiac artery of less than 180°, without showing stenosis or deformity; tumor contact with the common hepatic artery, without showing tumor contact with the proper hepatic artery and/or celiac artery; and tumor contact with the superior mesenteric vein and/or portal, without extending beyond the inferior border of the duodenum. Biological factors include potentially resectable diseases, based on anatomic criteria but with clinical findings suspicious for (but unproven) distant metastases or regional lymph nodes metastases, diagnosed by biopsy or positron emission tomography-computed tomography (PET-CT). Therefore, radiological templates should report features on the presence and degree of contact between the tumor and the vessels, such as irregularities of the vessel contours (including a “tear drop” deformity) or changes in caliber, since these are signs of vascular invasion [75]. Moreover, several additional findings, which are relevant for procedural planning, should be reported as the arterial variants and the origin of the right hepatic artery from the superior mesenteric artery (SMA) (Figure 5) [77,78].

In this context, SR could help and guide the radiologist, allowing them to identify all the significant features that could modify patient management [79,80,81,82,83,84].

**Figure 6:**
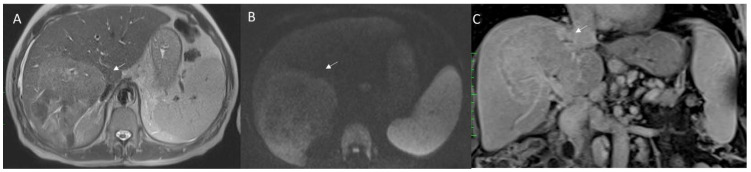
Cholangiocarcinoma patient, classified as LR-M according to LI-RADS, due to targetoid appearance (arrow) in T2-W (**A**) sequence, in (**B**) DWI, and (**C**) late phase of contrast study.

At the highest level, SR has all the previously mentioned features and uses a standardized language, based on a universally accepted lexicon [9,85,86]. A significant example in the field of breast imaging, is the breast imaging reporting and data system (BI-RADS), promoted by the American College of Radiology (ACR). BI-RADS includes a standardized lexicon for the description of breast imaging findings and their clinical management [15]. Like BI-RADS, in order to standardize the reporting and interpretation of imaging data, similar approaches have been introduced for different lesions, such as liver lesions, thyroid lesions, prostate lesions, and nodal evaluation [30]. Regarding HCC, the Liver Imaging Reporting and Data System (LI-RADS) represents a way of interpreting and reporting radiological findings that were obtained by CT or MRI, in patients at risk for this tumor. Despite the introduction of a new category, LR-M extends this system to other lesions, such as cholangiocarcinoma (Figure 6) and metastases. The American College of Radiology (ACR) supported the spread of LI-RADS to homogenizing the interpreting and reporting data of liver lesions. The diagnosis of hepatocellular carcinoma is due to the presence of important imaging features, which allow us to classify LI-RADS-3, LI-RADS-4, and LI-RADS-5, and to include arterial-phase hyperenhancement, tumor diameter, wash-out appearance, capsule appearance, and threshold growth. Ancillary features are features that can be used to change the LI-RADS classification. In MRI studies, ancillary features that support malignancy can be used to update the category of one or more categories, but not beyond LI-RADS-4 [30].

## 5. Open Questions: Radiologists and SR

The major international societies of radiology have supported the SR application [78,87,88,89].

The Radiological Society of North America’s (RSNA) reporting initiative has contributed to the dissemination of SR by developing and freely distributing hundreds of SR templates online at radreport.org. The Italian Society of Medical and Interventional Radiology (SIRM) suggested several templates that can be easily utilized by members of SIRM [90].

Despite the clear advances, SRs have not yet been used in the radiological workflow. The main motives are related to the absence of standardized, shared templates and the, again, marginal accessibility of software tools [91,92]. Moreover, other reasons revolve around changing practice habits and the radiologists’ fear of losing their style of communication to templated language.

The fundamental purpose of diagnostic radiology is to assist clinical care and assess patient outcomes through the acquisition, evaluation, and communication of radiological findings. The images are acquired and evaluated considering the clinical issue and are communicated in return to the clinicians and patients, offering a clear picture of the patient’s disease. Therefore, during radiologists’ work-up, it is possible to recognize the following two main phases: the first is related to the imaging interpretation, which involves the identification and recognition of the salient radiological findings. This phase is connected to diagnosis. The second, similarly crucial, is the transfer of the evaluated findings and the results to the referring clinicians and patients, suitably and clearly, in a report. Competence in one of these phases does not necessarily mean competence in the other. The communication of radiological data is essential, but how one arrives at that goal may vary according to diverse manners (structured reports, elegant language). It has been assumed that FTRs have progressed for the suitability of the report author, not for the reader [93,94,95,96,97,98,99,100,101,102]. The radiologist could suppose that their own narrative report is more useful and valuable, compared to SR. However, the reader may not approve. Structured reporting may reduce the personality in the description, and it may favor standardization—even if SR may retain several freedoms for the radiologist in the conclusion. Once the report section has included all relevant findings and observations, the radiologist should summarize their opinion in a conclusion, using free text [92]. Radiological SR is available for a growing number of patients and diseases. This may be an additional motivation for the radiologist to embrace SR, in particular to use quantified data for describing findings, rather than subjective qualitative reporting/interpretations, such as, “slight”, “mild”, or “significant” increase in volume. How much is “slight”, “mild”, or “significant”? It would be better simply to specify the size (volume) of a structure or lesion and its evolution in time [92].

## 6. Pitfalls

The SR has several established limitations, such as the possibility of oversimplification and the rigor of the model structure, as well as poor user compliance [92]. However, as explained above, considerable energy has been spent by experts and scientific societies for SRs in various clinical settings. Their use in radiology departments with high degrees of acceptance may not be complicated and adaptation to the individual characteristics of the department may be feasible and should be considered.

To this end, specific queries are open, comprising better integration with pre-existing RIS/PACS systems, the option to maintain several free text fields within templates (thus allowing some margin to communicate complex concepts in selected cases), as well as data related to the imaging protocol [92].

The integration of SR in the PACS offers significant advantages in communicating with the referring physicians, in particular in education centers and in communications with referring general physicians. Links to the descriptive data and the relevant images (size, arrows), and to the image-related or derived parameters can be easily integrated into the report [92].

## 7. Educational: Resident and Structured Report

Definite benefits for residents comprise the possibility to increase clinical knowledge, and to develop communication competence and efficacy [103].

According to the 2019 Diagnostic Radiology Milestones [104], each resident should “expertly use lexicons and structured reporting to offer precise and well-timed reports which do not necessitate modification”. Imaging reporting is an important resident skill set, since radiology reports are the most frequent method of radiologists’ communication. Residents should improve their communication method and become effective communicators [105]. SR optimizes the sharing of the main relevant data in clinical and radiological settings for liver cancer [46,50,86,104,106,107,108,109] and for rectal cancer [27,42,110,111,112], such as in the multidisciplinary treatment of several deep tumors [46,113,114,115,116,117,118,119]. The resident should be able to learn the specific data on the diagnostic tool to be adopted, such as the type of contrast medium and the way in which this drug could be used (simply, the enhanced phase of the contrast, versus dynamic acquisition). All these data, which are included in the SR in a predictable, reproducible, succinct, organized, and precise format, provide a framework for radiology trainees. The form and content of each SR could be utilized as a didactic tool, which is useful for students in assisting them to obtain their autonomous training [103].

## 8. Discussion and Conclusions

The key elements of quality and safety programs are to optimize communication and to address failures in communication. Unsuccessful communication is an important cause of medical errors within the radiology field [79]. A standardized lexicon that is used to express radiologists’ diagnoses may improve patient care, as the adoption and use of SR is an important method of enhancing communication. Although recently published data and several radiology societies [79,89,119,120,121,122] support SR as a required new standard for radiology reporting, with clear benefits over narrative reporting, radiologists prefer prose reporting, due to its flexibility and personalization. In addition, despite these incentives, there is not much data that objectively demonstrates that SR adds clinical value, over narrative reporting [10,123,124]. Moreover, not all SRs are designed similarly. Several of the published SRs are technique- or examination-based [125], and, though they are easy to use, they do not guide the radiologist during image evaluation, only providing fields to complete in a “structured” method. A common factor seen in most definitions, is that a SR should help the writers create their report, through either a predefined design, template, or checklist. Furthermore, SR only represents one set of computer tools, aimed at reducing variability and enhancing the clinical utility of formal radiology interpretations. Therefore, SRs should be created according to the third level of Weiss et al. [31], representing contextual reporting, precisely correlated to a particular clinical setting. In this way, SR provides content focused on the clinical questions, discusses appropriate differential diagnoses, and highlights pertinent positives and negatives in the data. In addition, to preserve radiologist freedom, these templates retain free-text sections. These reports are exclusively tailored to the diagnosis, they guarantee that all pertinent data are addressed in a checklist, and they educate trainees by providing a systematic approach for clinical interpretations. Furthermore, these models are based on standardized language and structures—qualities required to adhere to diagnostic–therapeutic recommendations and enrollment in clinical trials. These could reduce any ambiguity that may arise from unconventional language and allow for better communication between radiologists, clinicians, and patients.

An additional benefit is related to the possibility that SR could, theoretically, have a substantial role in data tracking and machine learning. Because these are disease-specific and structured, common features can be gathered from the reports so that computers can read and understand the content to improve radiologist workflow. In fact, in recent years, the application of artificial intelligence (AI) within radiology has intensified [13,37,105,106,107,108,109,110,111,112,113,114,115,116,117,118,119,120,121,122,123,124,125,126]. Although many research studies have been focused on imaging interpretation and classification, AI can support all areas of a radiological division, such as the use of learning algorithms for protocol selection, decreasing radiation doses, reducing acquisition times, and improving patient safety. In this context, it is easy to think that AI algorithms will certainly become an integral part of the radiology reporting systems. For example, an AI algorithm could automatically calculate the percentage change in size from the previous study, and automatically report whether there is a threshold growth in a certain observation, allowing a faster and more precise definition of the LI-RADS category. In addition, the AI algorithm could allow the linking of imaging results and other clinical data in order to achieve a personalized approach to the patient. In fact, when it comes to accessibility, radiologists’ reports are known to be an opulent source of research data, enabling automated data mining, which can help validate imaging biomarkers.

Structured reporting could eliminate the individuality of radiologists’ reports, allowing a standardized approach.

Structured reporting is thought to improve the consistency and reproducibility of radiological reports [125]. This could improve the readability and clarity of radiological reports, and could also facilitate data mining in clinical or research settings.

## Data Availability

Not applicable.
